# Short-Wavelength and Infrared Autofluorescence Imaging in Pachychoroid Neovasculopathy

**DOI:** 10.3390/vision9020038

**Published:** 2025-04-21

**Authors:** Norihiko Nakagawa, Takuya Shunto, Issei Nishiyama, Kohei Maruyama, Miki Sawa

**Affiliations:** 1 Department of Ophthalmology, Sakai City Medical Center, Osaka 593-8304, Japanissei2480@gmail.com (I.N.); mont.kohei@gmail.com (K.M.); 2 Department of Ophthalmology, Osaka University Graduate School of Medicine, The University of Osaka, Osaka 565-0871, Japan

**Keywords:** pachychoroid neovasculopathy, short-wavelength autofluorescence, infrared autofluorescence

## Abstract

Purpose: The purpose of this paper is to investigate the relationship between short-wavelength autofluorescence (SWAF) and infrared autofluorescence (IRAF) patterns in pachychoroid neovasculopathy (PNV) with serous retinal detachment (SRD). Methods: This study used an observational case series of 62 eyes of 58 consecutive patients diagnosed with symptomatic PNV from January 2019 and October 2021 at a single institution. SWAF and IRAF patterns were analyzed with disease chronicity, and autofluorescence changes in macular neovascularization (MNV) were assessed in two images. Results: SWAF patterns and the mean duration of symptoms were as follows: blocked (15 eyes, 24%), 1.0 months; mottled (8 eyes, 13%), 2.8 months; hyper (24 eyes, 39%), 5.0 months; hyper/hypo (10 eyes, 16%), 7.0 months; descending tract (5 eyes, 8%), 12.0 months (*p* < 0.01). IRAF patterns and the mean duration of symptoms were as follows: blocked (17 eyes, 27%), 1.0 months; hyper (22 eyes, 35%), 4.0 months; mixed/hyper dominant (9 eyes, 15%), 5.0 months; mixed/hypo dominant (9 eyes, 15%), 6.8 months; descending tract (5 eyes, 8%), 12.0 months (*p* < 0.01). Abnormal autofluorescence corresponding to MNV lesion was seen in 34 eyes (55%) with SWAF and 59 eyes (95%) with IRAF (*p* < 0.01). Conclusions: SWAF and IRAF show multiple patterns and are related to disease chronicity in symptomatic PNV. IRAF could be helpful in detecting the lesion of MNV.

## 1. Introduction

Pachychoroid neovasculopathy (PNV) is a clinical entity of type-1 macular neovascularization (MNV) with the morphological and functional abnormalities of thick choroid [1]. Recently, it has been widely accepted that pachychoroid spectrum disease, including central serous chorioretinopathy (CSC), pachychoroid pigment epitheliopathy, PNV, polypoidal choroidal vasculopathy, focal choroidal excavation, and peripapillary pachychoroid syndrome, shares pachychoroid features [2]. Retinal pigment epithelium (RPE) alteration or dysfunction can be seen in the eyes of patients with pachychoroid spectrum disease [3,4].

Fundus autofluorescence (FAF) is a non-invasive imaging technology that can show functional activity in the RPE of living eyes [5]. Short-wavelength autofluorescence (SWAF) is commonly used for the diagnosis of retinal diseases and monitoring of the disease progression in retinal dystrophy including retinitis pigmentosa, age-related macular degeneration, and CSC by detecting RPE changes associated with lipofuscin [6,7,8,9]. Infrared autofluorescence (IRAF), another wavelength, is also helpful to monitor the status of melanin [10,11]. However, the study using IRAF is limited compared to SWAF because of its low sensitivity in detecting melanin changes and the scarcity in the apparatus such as confocal scanning laser ophthalmoscope in clinical practice.

Several studies have demonstrated the association between FAF findings and disease severity in eyes with CSC [7,8,11]. On the contrary, FAF studies focused on PNV are limited because this is a newly categorized disorder. We postulate that FAF may provide new information on the pathologic process of symptomatic PNV. The aim of our study is to describe SWAF and IRAF findings in PNV and to elucidate the relationship between these imaging modalities.

## 2. Materials and Methods

### 2.1. Subjects

The medical records of the patients diagnosed as having PNV accompanied with serous retinal detachment (SRD) between January 2019 and October 2021 at a single institution (Eye Center, Sakai City Medical Center, Osaka, Japan) were reviewed retrospectively. Patients older than 65 years were excluded. Eyes were excluded if they had undergone any intraocular surgery, laser photocoagulation, or photodynamic therapy. Eyes with subretinal hemorrhage and polypoidal lesion were excluded. Eyes with bilateral involvement were analyzed individually.

The criteria for the diagnosis of PNV in this study were as follows: (1) MNV detected by FA and ICGA, (2) a shallow irregular RPE detachment at the site of MNV observed on OCT (type-1 MNV), and (3) pachychoroid features, namely, reduced fundus tessellation, no drusen or only pachydrusen, and the presence of dilated choroidal vessels below the type-1 MNV. Central choroidal thickness and choroidal vascular hyperpermeability were not included among the criteria for PNV.

### 2.2. Ophthalmic Examinations

All patients underwent comprehensive ophthalmologic examinations including best-corrected visual acuity (BCVA) using a Landolt C chart, slit-lamp biomicroscopy, fundus photography (TRC-50DX, Topcon, Tokyo, Japan), spectral domain optical coherence tomography (SD-OCT), enhanced depth imaging OCT (EDI-OCT) (Spectralis OCT2, Heidelberg Engineering, Heidelberg, Germany), fluorescein angiography (FA), indocyanine green angiography (ICGA), and fundus autofluorescence (FAF) imaging (Spectralis Heidelberg Retina Angiograph [HRA] 2, Heidelberg Engineering, Heidelberg, Germany). OCT angiography (OCTA) (Spectralis Heidelberg Retina Angiograph [HRA] 2, Heidelberg Engineering, Heidelberg, Germany) was performed in a limited number of patients.

FAF measurements were performed using an excitation light with a wavelength of 488 nm and detection filter > 500 nm in SWAF, and 787 nm and >800 nm in IRAF, respectively. An automatically averaged image was acquired from a series of images, thereby providing a more detailed image with enhanced contrast. All FAF examinations were performed before FA and ICGA.

EDI-OCT scans in the horizontal and vertical lines were made through the center of the fovea. In addition, horizontal raster scans were performed at the extrafoveal area to detect subretinal fluid in the posterior pole. The central choroidal thickness was defined as the distance between Bruch’s membrane and the chorioscleral interface at the fovea in EDI-OCT images and manually measured by the inbuilt caliper of the software (Heidelberg Eye Explorer).

The patients were interviewed about the onset of subjective symptoms such as visual loss and metamorphopsia to estimate the disease duration.

### 2.3. Classification of FAF Images

Abnormal FAF findings were defined as either increased or decreased FAF signal compared with the normal FAF, outside of SRD lesions. We evaluated FAF patterns and focal AF change corresponding to MNV.

**Figure 1:**
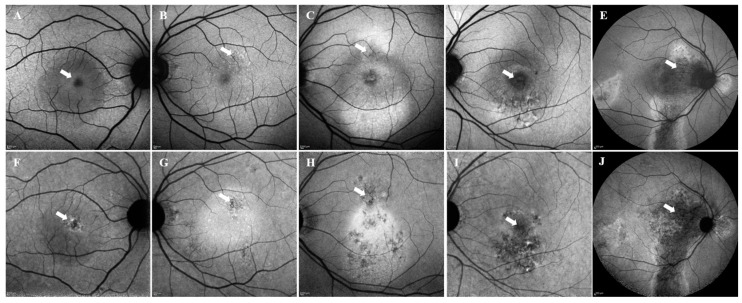
Representative images of SWAF and IRAF patterns. SWAF patterns are shown in (**A**–**E**). (**A**) uniformly decreased FAF intensity (blocked), (**B**) dot-like increased FAF intensity with normal background (mottled), (**C**) increased FAF intensity (hyper), (**D**) mixture of increased and decreased FAF intensity (hyper/hypo), (**E**) downward leading swathe of FAF changes originating from the posterior pole to extend beyond the inferior arcade (descending tract). IRAF patterns are shown in (**F**–**J**). (**F**) blocked, (**G**) hyper, (**H**) hyperautofluorescence > 50% (mixed/hyper dominant), (**I**) hypoautofluorescence > 50% (mixed/hypo dominant), (**J**) descending tract. (**A**) and (**F**), (**B**) and (**G**), (**C**) and (**H**), (**D**) and (**I**), (**E**) and (**J**) belong to the same patient, respectively. White arrows indicate focal AF at the site of MNV.

We classified FAF patterns based on previous observational studies [12,13]. SWAF was classified into five patterns: blocked, mottled, hyper, hyper/hypo, or descending tract. A pattern was defined as follows: blocked AF showed uniform changes in decreased autofluorescence; mottled AF showed a grainy or coarse region of increased autofluorescence compared to the surrounding background; hyper AF showed predominantly increased autofluorescence; hyper/hypo AF showed a mixed form of increased and decreased autofluorescence; and descending tract showed a downward leading swathe of decreased autofluorescence originating from the posterior pole to extend below the inferior arcade, as described previously [8]. Similarly, IRAF was classified into five patterns as blocked, hyper, mixed/hyper dominant, mixed/hypo dominant, or descending tract. Mixed/hyper dominant or mixed/hypo dominant was defined as hyperautofluorescence or hypoautofluorescence lesions accounting for more than half of the abnormal FAF areas. The representative images of each FAF pattern are shown in Figure 1. Focal SWAF and IRAF change in MNV lesion was divided into normal (iso) and abnormal. Abnormal FAF change was subdivided into four patterns as hypo, hyper, and mixed (hyper/hypo) AF compared to the surrounding background.

Two individual ophthalmologists (N.N. and M.S.) graded the FAF images based on the predominant pattern in the area of subretinal fluid in the posterior pole and MNV lesion. When the opinions of the observers were different, a third observer’s decision (T.S.) was considered.

### 2.4. Statistical Analysis

All statistical analyses were performed using the JMP version 14 software (SAS Institute, Cary, NC, USA). Conversion of decimal visual acuity to the logarithm of the minimum angle of resolution (logMAR) was used for statistical analyses. The disease duration and age between FAF patterns were compared by Kruskal–Wallis test with a post hoc Steel–Dwass test. McNemar’s test was used to evaluate differences in the detection of MNV between SWAF and IRAF. Interobserver variability was determined with k statistics. A *p*-value < 0.05 was considered statistically significant.

## 3. Results

The subjects of this study were 62 eyes of 58 patients with symptomatic PNV. The patient age ranged from 33 to 64 (mean ± SD, 49 ± 7) years, with 47 men (81.0%) and 11 women (19.0%). The median duration of symptoms was 3.5 (range, 0.17–60.0) months. Four eyes had previously undergone intravitreal injection; however, subretinal fluid did not resolve after the treatments.

**Figure 2:**
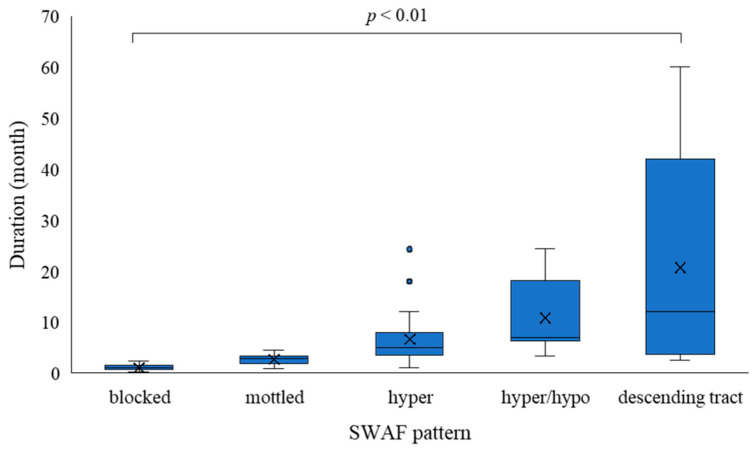

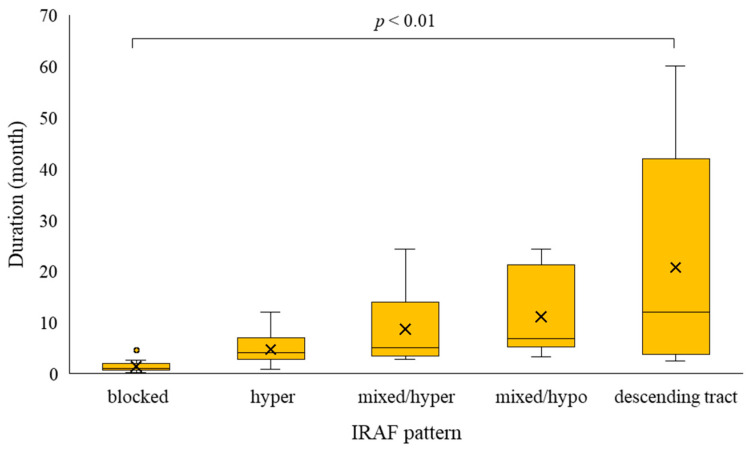
Disease duration according to SWAF and IRAF patterns. Abbreviation: SWAF, short-wavelength autofluorescence; IRAF, infrared autofluorescence. Kruskal–Wallis test.

**Figure 3:**
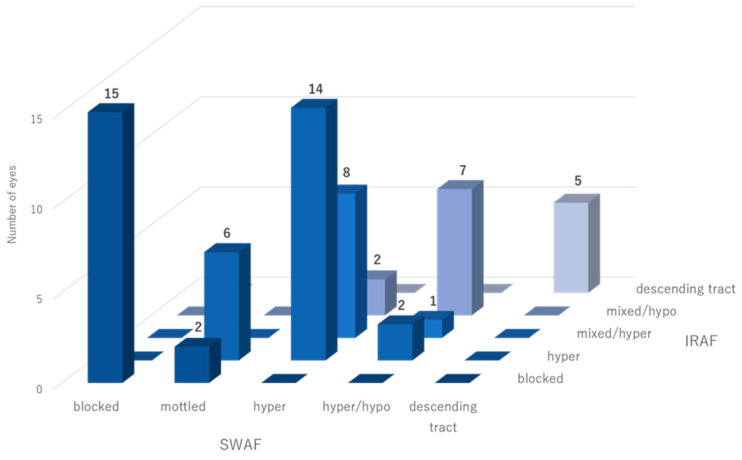
Relationship between SWAF and IRAF patterns. Abbreviation: SWAF, short-wavelength autofluorescence; IRAF, infrared autofluorescence.

The numbers of eyes in each pattern and the mean duration of symptoms were as follows: in the SWAF images, blocked AF (15 eyes, 24%): 1.0 months; mottled AF (8 eyes, 13%): 2.8 months; hyper AF (24 eyes, 39%): 5.0 months; hyper/hypo AF (10 eyes, 16%): 7.0 months; and descending tract (5 eyes, 8%): 12.0 months. In the IRAF images, blocked AF (17 eyes, 27%): 1.0 months; hyper AF (22 eyes, 35%): 4.0 months; mixed/hyper dominant AF (9 eyes, 15%): 5.0 months; mixed/hypo dominant AF (9 eyes, 15%): 6.8 months; and descending tract (5 eyes, 8%): 12.0 months (Figure 2). The interobserver variability was as follows: *k* = 0.76 (SWAF) and *k* = 0.81 (IRAF). The relationship between SWAF and IRAF is shown in Figure 3. There was a significant difference in the disease duration according to the FAF patterns. In SWAF, the blocked pattern showed the shortest duration, followed by mottled AF, hyper AF, hyper/hypo AF, and descending tract (*p* < 0.01). In IRAF, the blocked pattern showed the shortest duration, followed by hyper AF, mixed/hyper dominant, mixed/hypo dominant, and descending tract (*p* < 0.01). There was no significant difference in patient’s age according to FAF patterns (Table 1).

**Figure 4:**
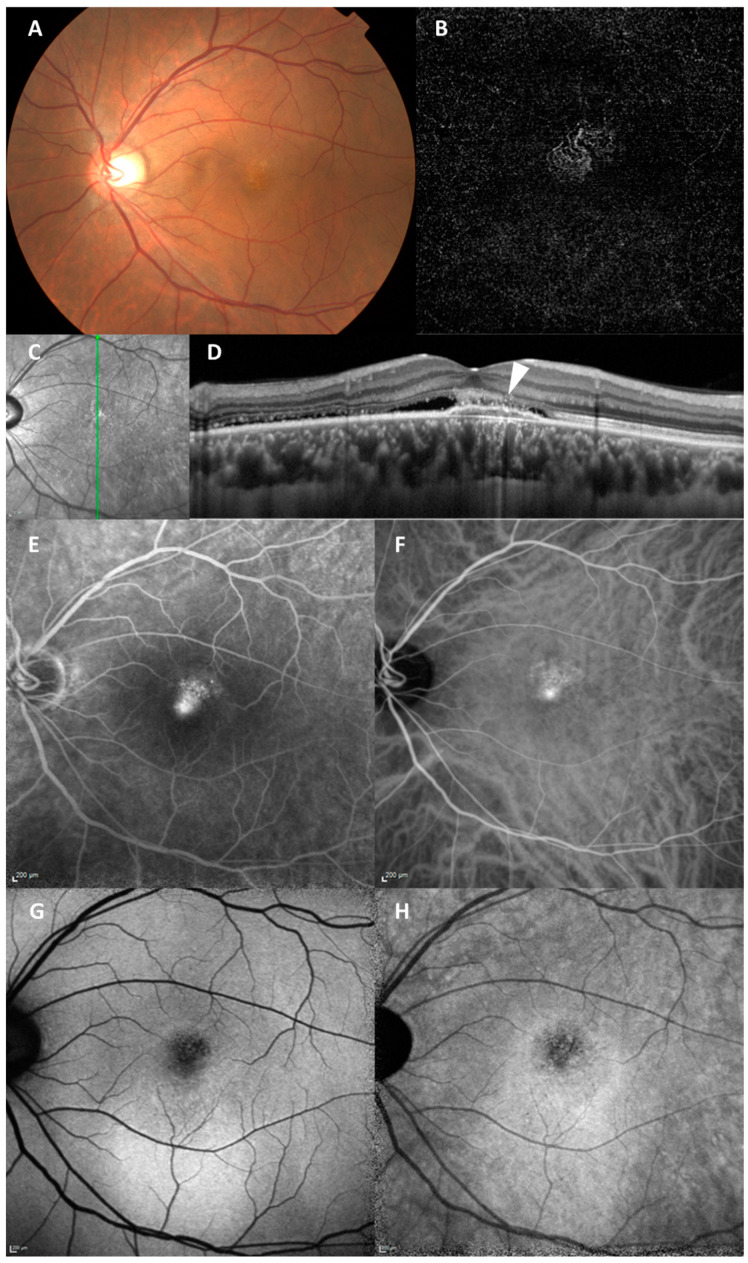
A representative case of PNV with multimodal imaging. A 47-year-old Asian male with treatment-naïve PNV. (**A**) Color fundus photograph showing reduced fundus tessellation with no drusen. (**B**) OCTA showing MNV. (**C**) Near-infrared fundus image with the vertical green line through the fovea corresponding to the B-scan on the right. (**D**) EDI-OCT showing SRD and RPE elevation consistent with type-1 MNV (arrowhead). (**E**) FA showing hyperfluorescence indicating leakage from MNV. (**F**) ICGA showing hyperfluorescence indicating MNV. (**G**) SWAF showing hyper AF pattern and hypo AF in the area with MNV. (**H**) IRAF showing the hyper AF pattern and hypo AF in the area with MNV.

For focal AF change corresponding to MNV lesion, the numbers of the eyes were as follows: in SWAF images, normal AF (28 eyes, 45%) and abnormal AF (34 eyes, 55%); in IRAF images, normal AF (3 eyes, 5%) and abnormal AF (59 eyes, 95%). A total of 27 of 28 (96%) eyes with normal SWAF showed abnormal IRAF. IRAF was significantly more likely to detect abnormal AF compared to SWAF (*p* < 0.01). For more detail on abnormal AF change, the numbers of each pattern were as follows: in SWAF images, hypo AF (24 eyes, 39%), hyper AF (3 eyes, 5%), and mixed AF (7 eyes, 11%); in IRAF images, hypo AF (46 eyes, 74%), hyper AF (3 eyes, 5%), and mixed AF (10 eyes, 16%). The interobserver variability was as follows: *k* = 0.68 (SWAF) and *k* = 0.82 (IRAF). A representative case of PNV with multimodal imaging is shown in Figure 4.

## 4. Discussion

We compared clinical findings about SWAF and IRAF imaging to study the characteristics of the eyes with symptomatic PNV. Both SWAF and IRAF patterns show the time-dependent changes from blocked, hyperautofluorescence to hypoautofluorescence. Focal AF change corresponding to PNV lesion is more detectable in IRAF than SWAF as abnormal autofluorescence change. Although IRAF imaging is less common than SWAF in clinical practice, IRAF can be tolerated as a supplemental approach to predict the disease chronicity and to locate MNV.

In the concept of pachychoroid spectrum disease, PNV consisting of type-1 MNV is possibly secondary to CSC [1]. Subretinal fluid in CSC has a potential of spontaneous resolution in the acute phase. However, in the chronic phase, such as chronic CSC or after progression to PNV, subretinal fluid is expected to be prolonged. Physicians would need information about chronicity when considering the optimal timing of treatment such as photodynamic therapy or anti-vascular endothelial growth factor therapy to avoid further visual acuity deterioration. The disease duration is mainly judged on the subjective information including the patients’ interview. In addition, non-invasive examinations such as FAF imaging would be clinically appropriate to assess the chronicity.

In the current study, the chronicity of SRD was evaluated based on FAF patterns. In the SWAF image, we followed the previous report about CSC by Han et al. including blocked, mottled, hyper, hyper/hypo, and descending tract [12]. Similar FAF pattern changes over time were seen in the eyes with PNV, probably because CSC and PNV shares the disease features. However, in IRAF images, there were few previous studies on the patterns and the duration of symptoms even in CSC [13]. Therefore, we modified the classification of IRAF pattern based on a previous report by Sekiryu et al. [13], including blocked, hyper, mixed/hyper, mixed/hypo, and descending tract which would be comparable to the classification of SWAF. Similarly to the SWAF patterns in our study, IRAF also showed a significant difference in the duration of symptoms between the patterns. Another study about FAF images with two wavelengths for the subjects with CSC showed the usefulness of estimating the disease duration and retinal damage [14]. In addition to routine SWAF images, IRAF images would be helpful to estimate the disease chronicity in PNV. The *k*-values of interobserver variability in the current study were 0.76 and 0.81 in SWAF and IRAF, respectively. Considering the *k*-value was 0.76–0.86 in the previous report of SWAF patterns by Han et al. [12], our classification of SWAF and IRAF might be suitable.

The formation of FAF changes was not simultaneous in SWAF and IRAF. The pattern on AF images in the fovea of case 2 (Figure 1B,G) and 3 (Figure 1C,H) showed more discrepancy compared to other cases (Figure 1). We speculated the differences may be due to macular pigment, which has light-absorbing properties in the spectrum range of 400 nm to 540 nm, and this property affects SWAF but not IRAF [15]. Sasamoto et al. reported that macular pigment was reduced in the eyes with chronic CSC, but not in the eyes with acute CSC [16]. We hypothesized that in the relatively short time from the onset including case 2 and 3, macular pigment may be less damaged, thereby making SWAF and IRAF appear distinctively different in addition to fluorophore difference in each imaging [9].

The alterations in SWAF corresponding to the site of MNV in neovascular age-related macular degeneration (nAMD) were previously reported [6]. Classic MNV often presents hypoautofluorescence and occult MNV shows both increased and decreased autofluorescence. However, studies on the characteristics of MNV using IRAF are scarce. PNV consisting of type-1 MNV, which is thought to be equivalent to occult MNV, would be expected to show increased and decreased autofluorescence changes based on previous report about nAMD [6]. However, in this study, SWAF images showed iso (normal)-AF at the site of MNV in half of the eyes. On the contrary, IRAF images demonstrated hypo-AF in three quarters of the eyes, and half of the eyes with hypo-IRAF showed no FAF change in SWAF images. One possible reason for this difference is that PNV primarily involves pachychoroid and RPE damage might occur sequentially [1,2,3,4]. Melanin detected by IRAF is from both RPE and choroid, and lipofuscin detected by SWAF is only from RPE [17]. To evaluate the subclinical change associated with pachychoroid as primary lesion, IRAF monitoring melanin might have an advantage compared to SWAF. Another imaging technique, OCTA, is advantageous when detecting type-1 MNV associated with PNV, which is relatively smaller than that of nAMD [18]. Based on the high rate of focal FAF abnormality in IRAF imaging, IRAF may be tolerated to point the location of MNV in OCTA examinations. However, detection of focal FAF abnormality corresponding to MNV might be limited in widespread FAF abnormalities such as a descending tract pattern.

Moreover, the difference in sensitivity can be attributable to the autofluorescence of the macula in two modalities. The SWAF image of the fovea is naturally hypo-AF due to absorption of the blue excitation light by the macular xanthophyll pigments. On the contrary, the fovea on IRAF appears brighter than the parafoveal region due to the higher content of melanin in this region and the lack of IRAF light filtering by xanthophylls [19]. MNV tends to grow in the center of the fovea. The brighter fovea in IRAF provides greater contrast for MNV, and therefore, MNV can be more detectable in IRAF than SWAF [20]. Another possible mechanism to explain the differing sensitivity is that melanin degradation precedes decreased lipofuscin level in the MNV lesion. Recently, the advanced imaging technique, polarization-sensitive OCT, was developed and can be useful to evaluate melanin [21,22]. A future study using multimodal imaging including polarization-sensitive OCT might clarify this point.

## 5. Limitation

The present study had several limitations. First, the sample size was small in each FAF pattern because of the classification into five groups. Second, the estimation of disease chronicity was dependent on the subjective recall of the patients. Basically, the objective assessment of disease onset is impossible. Third, our study lacked quantitative assessment of the FAF images. However, the process of normalized gray value was not effective because the FAF images were obtained as automatically averaged multiple images. Fourth, the classification of FAF pattern has not been standardized across different studies. Further investigations in larger sample size and in multicenter collaborative research would be necessary to identify the optimal classification of FAF in PNV.

## 6. Conclusions

FAF imaging using both SWAF and IRAF could be a useful tool for objective assessment of chronicity in symptomatic PNV. In addition, the present study suggests that IRAF can be more capable of detecting the lesion of MNV than SWAF due to the contrast differences.

## Figures and Tables

**Table 1:** Difference in patient characteristics between fundus autofluorescence patterns.

**SWAF**	blocked	mottled	hyper	hyper/hypo	descending tract	*p* value
Number (%)	15 (24)	8 (13)	24 (39)	10 (16)	5 (8)	
Duration of symptom (months)	1.0 (0.7–1.5)	2.8 (1.8–3.4)	5.0 (3.5–8.0)	7.0 (6.3–18.1)	12.0 (3.8–42.0)	<0.01
Age (years)	48.4 ± 7.6	46.8 ± 7.6	50.8 ± 6.6	50.3 ± 8.9	49.8 ± 11.6	0.71
**IRAF**	blocked	hyper	mixed/hyper	mixed/hypo	descending tract	*p* value
Number (%)	17 (27)	22 (35)	9 (15)	9 (15)	5 (8)	
Duration of symptom (months)	1.0 (0.7–3.0)	4.0 (2.8–8.8)	5.0 (3.4–14.0)	6.8 (5.3–21.3)	12.0 (3.8–42.0)	<0.01
Age (years)	48.5 ± 7.2	48.4 ± 8.1	50.0 ± 6.3	53.9 ± 6.3	49.8 ± 11.6	0.41

Data were presented as median (IQR) in the duration of symptoms and mean ± standard deviation in the age. Abbreviation: SWAF, short-wavelength autofluorescence; IRAF, infrared autofluorescence. Kruskal–Wallis test.

## Data Availability

The data that support the findings of this study are available from the corresponding author upon reasonable request.
